# The calcareous brown alga *Padina pavonica* in southern Britain: population change and tenacity over 300 years

**DOI:** 10.1007/s00227-015-2805-7

**Published:** 2016-02-10

**Authors:** Roger J. H. Herbert, Lisha Ma, Anne Marston, William F. Farnham, Ian Tittley, Richard C. Cornes

**Affiliations:** Faculty of Science and Technology, Department of Life and Environmental Sciences, Bournemouth University, Talbot Campus, Fern Barrow, Poole, Dorset BH12 5BB UK; Isle of Wight Local Records Centre, Seaclose Offices, Fairlee Road, Newport, Isle of Wight PO30 2QS UK; Institute of Marine Sciences, School of Biological Sciences, Portsmouth University, Ferry Road, Eastney, Portsmouth, PO1 2DY UK; The Natural History Museum, Cromwell Road, London, SW7 5BD UK; Climatic Research Unit, School of Environmental Sciences, University of East Anglia, Norwich, UK

## Abstract

Understanding long-term persistence and variability in species populations can help to predict future survival, growth and distribution; however, sustained observations are exceedingly rare. We examine and interpret a remarkable record of the calcareous brown alga *Padina pavonica* (Phaeophyceae) at its northern limit on the south coast of England (50°N, 1–3°W) from 1680 to 2014, which is probably the longest compilation and review of any marine algal species. Over this period, which extends from the middle of the Little Ice Age to the present, there has been considerable variability in temperature and storminess. We identified a significant number of site extinctions in the second half of the nineteenth century, which coincided with cooler conditions and stormier weather. To interpret these changes, we measured recruitment, growth and production of tetraspores at sheltered and exposed sites in 2012–2014, years which had low and high spring temperatures. Potential spore production was greater at the sheltered site due to a longer growing period and survival of larger fronds. Delayed growth in the cooler spring resulted in smaller fronds and lower potential production of tetraspores by early summer. Yet in the warmer year, rapid initial growth caused higher sensitivity to damage and dislodgement by summer storms, which also limited potential spore production. Antagonistic responses to multiple stressors and disturbances make future predictions of survival and distribution difficult. Fronds of *Padinapavonica* are sensitive to both temperature and physical disturbances, yet vegetative perennation appears to have enabled population persistence and explained the longevity of remaining populations.

**Electronic supplementary material:**

The online version of this article (doi:10.1007/s00227-015-2805-7) contains supplementary material, which is available to authorized users.

## Introduction

Understanding species response to environmental change is becoming increasingly important in predicting and mitigating impacts on biodiversity. Species may become extinct if they fail to adapt to the changing environment, acclimatise to the new conditions or maintain colonisation processes such as recruitment and dispersal (O’Conner et al. 2012). In regions that are experiencing warming and variance in the frequency and intensity of climatic events, better knowledge of species life-history strategies and sensitivity to meteorological stressors and disturbances may help predict future survival and distribution. Here, we use ‘stress’ to refer to mechanisms that negatively impact physiology, growth and reproduction and ‘disturbance’ refers to physical processes that may cause damage and removal of organisms (Grime 1977; Airoldi 2003). Examination of species’ response to a wide range of environmental variability (and hence multiple stressors and disturbance) ideally requires a long time-series, but sustained observations over more than a few decades are rare (Hawkins et al. 2013; Mieszkowska et al. 2014). Time-series analysis can be very valuable as knowledge of species’ ability to cope with past stress and disturbance can be a useful predictor of future survival and growth (Padilla-Gaminõ and Carpenter 2007). Algal turf and erect species are fundamentally important to ecosystem function in shallow seas (Harley et al. 2006, 2012), yet long-term studies on responses of marine algae to environmental change are particularly scarce (Harley et al. 2012; Wernberg et al. 2012), though see Yesson et al. (2015) for large brown seaweeds. The performance of seaweeds is significantly influenced by temperature (Eggert 2012), and northern biogeographical boundaries of subtropical and warm-temperate algal species are usually set by lethal winter temperatures or low summer temperatures that prevent reproduction (Breeman 1988). Yet other direct or indirect impacts of climate may have influence on populations and could limit future growth. For example, sediment smothering and abrasion on rocky shores due to storm events can affect species survivorship, composition and diversity (Airoldi 1998, 2003; Vaselli et al. 2008), growth, reproduction and regeneration (Umar et al. 1998), competitive outcomes (Jenkins et al. 2004; Kraufvelin and Salovius 2004; Milazzo et al. 2004) and interactions with grazers (Jenkins et al. 1999).

Although mobile species may respond quickly to unfavourable conditions by migration, sessile species with generally poor dispersal capability such as algae require strategies for survival over longer periods of suboptimal environmental conditions (Dixon 1965; Harley et al. 2012). Algal persistence in disturbed areas has been attributed to life-history strategies that include perennation, vegetative propagation (Dixon 1965; Airoldi 1998), multiple releases of spores and a combination of these strategies (Eriksson and Johansson 2005).

To investigate long-term responses of marine algae to stress and disturbance, we examined a unique time-series of observations and herbarium records (1680–2014) of *Padina pavonica* (L.) Thivy (Phaeophyceae, Dictyotales) in southern Britain, which must be the longest compilation and review of any marine algal species. *Padina* is one of the only two genera of calcified brown algae, and *P. pavonica* is the type species reported from the North-east Atlantic European coast, South Atlantic, Indian and Pacific Oceans, the Mediterranean (Guiry and Guiry 2014) and currently reaches its northern geographical limits in the British Isles. In many parts of the Mediterranean, *Padina* can be a prominent component of algal assemblages and occurs at depths to 60 m (Price et al. 1979; Guiry and Guiry 2014). However, on the Atlantic coast of Europe and in the English Channel the species is found mainly in pools on rocky shores consisting of soft substrata and only occasionally in the shallow infralittoral (~1 m). These shore platforms are usually backed by rapidly receding sandstone and mudstone cliffs, and the species is often found in close proximity to clay, silt or sandy sediments.

A previous analysis of the distribution of *P. pavonica* concluded that there had been a range contraction of the species in Britain and northern Europe (Price et al. 1979). It has been suggested that shifts in sediment distribution, caused by storm events and coastal erosion, could have contributed to the decline of *P. pavonica* in some parts of the British Isles (Price et al. 1979). An examination of the historical record in relation to temperature is important, as is consideration of the species reproductive traits. *Padina pavonica* has a haplodiplontic isomorphic life cycle, yet throughout its range gametophytic plants have been rarely reported (Carter 1927; Price et al.^1979^; Gómez-Garreta et al. 2007). However, the production of asexual tetraspores is more commonly observed and has been recorded in populations on the south coast of England during summer months (Carter 1927; Price et al. 1979). Vegetative perennation and propagation are also thought important for maintaining extant populations (Price et al. 1979). The importance of these different mechanisms has not previously been studied, nor has there been investigation into the factors that limit the latitudinal distribution of the species and its temporal variability at the edge of its range. There is current interest and concern about the impact of ocean acidification on calcareous seaweeds, including *Padina pavonica* (Johnson et al. 2012; Betancor et al. 2014; Gil-Díaz et al. 2014; Celis-Plá et al. 2015), so it is important to understand how these species respond to a variety of other stressors to predict long-term population change.

To support interpretation of the times-series data, we also investigated the species short-term sensitivity to sea temperature, storminess and sand-smothering. We discuss and interpret the time-series and experimental data in the context of life-history traits and predict whether future environmental change is likely to favour or restrict population growth and range expansion.

We test the following hypotheses:

1. Cool and stormy conditions in the mid-late nineteenth century were responsible for the regional decline of *Padina pavonica* at the northern periphery of its range.
2. Production of tetraspores and fecundity of *P. pavonica* will increase in association with higher spring and summer temperatures;
3. Growth and fecundity of *P. pavonica* are negatively affected by physical disturbance and sand-smothering.

## Materials and methods

### Region of investigation

**Fig. 1:**
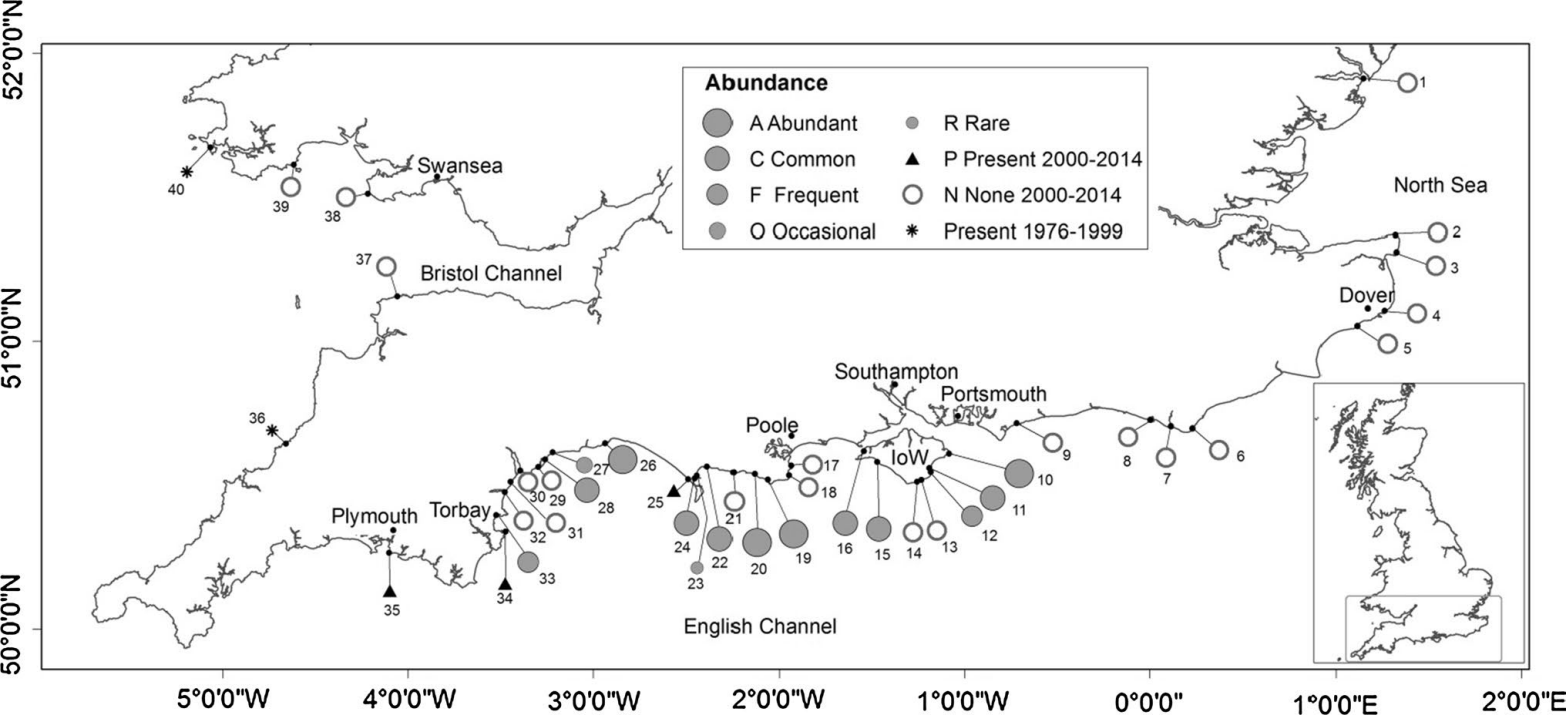
Distribution and abundance of *Padina pavonica* in southern Britain. *Symbols* show the maximum recorded abundance at each site over the period 2000–2014 and records from other localities subsequent to the previous main survey (Price et al. 1979). To our best knowledge, there have been no records north of this region subsequent to those in Price et al. (1979). The abundance scale and site reference numbers are given in Tables 1 and 2, respectively

Investigations were conducted on the coast of southern England (50°N, 1–3°W) where the majority of historical records of *Padina pavonica* in the British Isles have been obtained (Price et al. 1979; Fig. 1). The region straddles warm-temperate (Lusitanian) and cold-temperate (Boreal-arctic) biogeographical provinces (Forbes 1858; Hiscock 1998) and is characterised by shores of southern aspect, varied exposure and substratum. Shore platforms mainly consist of soft sedimentary rocks. Tidal range in the centre of the region is 2 m, increasing to 6 m in both the western and eastern Channel. Salinity on the open coast is 34–35 psu. There is now a large commercial and tourist infrastructure that includes major ports at Plymouth, Poole, Southampton, Portsmouth and Dover.

To investigate relationships between historical and current records of *P. pavonica* and sea temperature, the 1850 to 1870 median monthly sea surface temperature (SST) anomalies (1960–1991 baseline) for the 5 × 5 degree grid cell centred on 52.5 N, 2.5 W were extracted from the HadSST3.1.1.0 data set (Kennedy et al. 2011a, b). From 1871 to 2014, monthly SST anomalies (1960–1991 baseline) were calculated from data extracted from HadISST1 (Rayner et al. 2003) for grid cell 50–51N, 1–2W. Presently, mean summer (June–August) offshore sea surface temperature (SST) is 16–18 °C (Rayner et al. 2003). Yet temperatures within rock pools can be elevated to over 20 °C, so monthly inshore SST data were also obtained from the Bramblemet automatic weather station (Solentmet Support Group 2015) located in the Solent 19 km north-west of Bembridge (50°47′.41N, 1°17′.15W).

The longest time-series analysis of storm activity for the region was obtained for the North Sea using mean sea-level pressure (MSLP) data recovered as part of the EMULATE project (Cornes and Jones 2011). Zonal geostrophic wind speed components were calculated for grid squares over the domain 55°–60°N, 0°–5°E. The summer storm index (June–August) was considered more relevant to the survival of growing plants than the winter index when the fronds/thalli are absent.

### Time-series analysis

Historical records (1680–1978) of *Padina pavonica* from the south coast of England were mostly extracted from Price et al. (1979). This reference contains a thorough and exhaustive evaluation of all known manuscripts, museum records and herbarium specimens of *P. pavonica* throughout Europe. Criteria used to evaluate evidence of authenticity and likelihood of occurrence at a site were based on the quality of herbarium specimens and accompanying information, whether specimens were attached or drift, recorded habitat, knowledge of the biology and ecology of the species and the collecting habits, travels and activities of observers. Specimens of *P. pavonica* are morphologically very distinct and confusion with other species is unlikely, so the validity of historical records is relatively high. Apart from occasional anecdotal description of abundance, these are qualitative records of date and species presence at a locality and in some cases its absence during particular years. Only primary records from specimens in herbaria or manuscripts with a record of location and year and accepted as authentic by Price et al. (1979) were included in this analysis. A few additional records were obtained from museums and Biological Records Centres. Where localities were in very close proximity (1–2 km), these were often combined as a single ‘site’ and referred to the nearest ‘town’ or ‘Bay’ shown on current maps (Ordnance Survey 1:25,000). Further details on localities within sites are provided in ESM Table 1. The time-series record of *P. pavonica* at ‘Sites’ in specific ‘Years’ combined records from Price et al. (1979), other historical records and subsequent survey data to 2014. Sites where historical records existed and there had been no re-discovery between 2000 and 2014 are categorised as currently ‘lost’.

### Surveying current distribution

To update the species distribution and time series of Price et al. (1979), a new field survey was conducted between 2000 and 2014 at sites shown in Fig. 1. These localities had extant populations in the 1960s and 1970s, earlier populations (Price et al. 1979) or included other suitable habitat in which the species had not previously been recorded. All shores were surveyed at low spring tides during the months July–September when fronds are mature. Where possible, the presence of infralittoral populations was also investigated by snorkelling. Each site was surveyed for at least 30 min by the same persons on multiple occasions over the 5-year survey period. When first encountered, population abundance was measured by replicate timed searches of 5 min to record the number of separate ‘clumps’ of *P. pavonica.* A clump refers to a group of fronds >20 cm from the nearest isolated group of fronds and abundance was classified according to the scale in Table 1, modified from the abundance scale in Crisp and Southward (1958). Information on other current and historical records of *P. pavonica* from within and outside the main study area, including herbarium material, was obtained by contacting museums, Biological Records Centres and searching the National Biodiversity Network Gateway (NBN 2014). These records were evaluated prior to inclusion within the database.

**Table 1:** Scale used to assess abundance of *Padina pavonica* at survey locations

A	Abundant: >100 clumps per 5-min search; cover frequently exceeds 25 %
C	Common: 51–100 clumps per 5-min search; cover occasionally exceeds 25 %
F	Frequent: 11–50 clumps per 5-min search
O	Occasional: 2–10 clumps per 5-min search
R	Rare: 1–11 clumps in 30-min search
N	None found in 30-min searching

A ‘clump’ refers to an isolated group of fronds >20 cm from another group of fronds. Timed searches were carried out by a single fieldworker. The percentage cover values refer to estimates within a gridded 0.25 m^2^ quadrat placed over clumps

### Responses to sea temperature and physical disturbances: growth and reproduction

To help interpret the time-series and distribution data, responses to inter-annual variation in temperature and physical disturbances were investigated in existing populations of *P. pavonica* at sheltered and exposed sites at Bembridge, Isle of Wight (Fig. 1; Table 2 site no. 10). The two sites are 1 km apart, and both populations are situated between High Water Neap (HWN) tide and Mean Tide Level (MTL). There are no local streams or rivers that might cause significant reductions in shore salinity. The exposed ledge (Ethel Point) is immediately below a sandy beach (median particle size diameter 250 µm) that has frequently smothered the shallow rock pools to depths of 5–20 cm for varying duration and extent over the past 30 years (author’s observations). Following the complete smothering of the exposed ledge between May 2012 and May 2013, the population was monitored and compared with the population at the sheltered site (Colonels Hard).

**Table 2:**
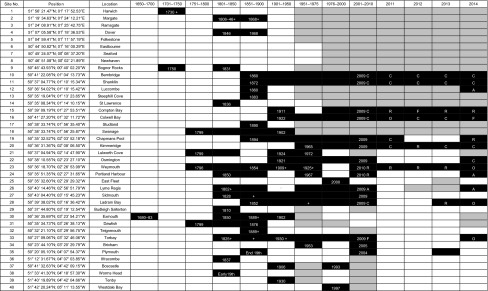
Longevity of site record of *Padina pavonica* in southern Britain. For locations of numbered ‘sites’, see Fig. 1

Black indicates presence during period shown. Grey indicates periods when *Padina* was not found in searches. Dates are shown to indicate first, last records or single record for the period. Recent records of ‘Abundance’ (A–R) are as shown in Table 1. Years when anecdotal historical records and accounts indicate *Padina* was ‘common’ or ‘abundant’ are shown as ‘+’

**Table 3:** Welch’s ‘*t*’ test results indicating significance of differences between mean values of frond length at sheltered (*CH* Colonels Hard) and exposed (*EP* Ethel Point) sites at Bembridge, measured during summer months in 2013 and 2014

Year	Month	Mean (mm) CH	Mean (mm) EP	*t*	*df*	*p*
2013	July	29.95	16.85	16.29	169.56	<0.001
	August	45.14	34.42	10.60	200	<0.001
	September	57.71	46.10	5.74	80.71	<0.001
2014	July	35.24	39.35	3.10	172.92	0.002
	August	36.54	33.79	2.24	393.14	0.016
	September	48.06	36.76	8.48	226.49	<0.001

### Growth and recruitment

**Fig. 2:**
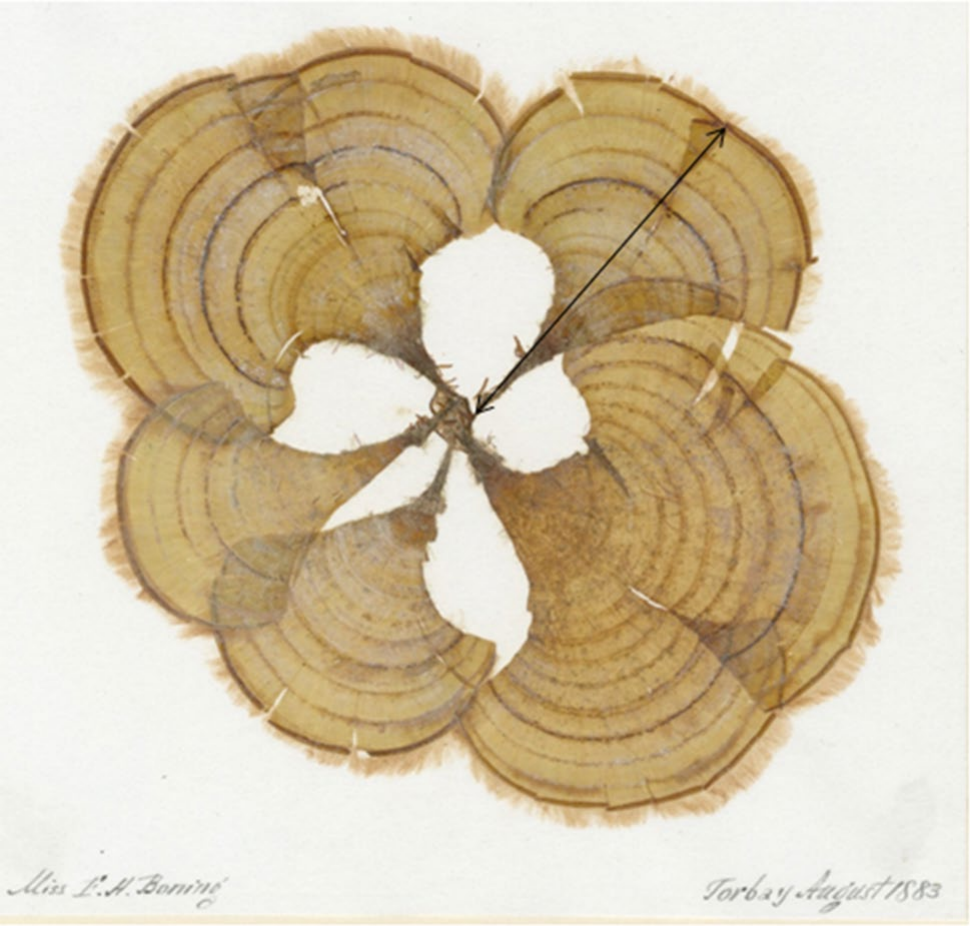
Herbarium specimen of *Padina pavonica* collected from Torbay, south coast of England, dated August 1883 (specimen registration number BM001063327, The Natural History Museum, London). The *black arrow* shows the measured parameter ‘frond length’ (50 mm)

Cover, clump density and recruitment at both sites were measured in 20 random quadrats (0.25 m^2^) placed along a transect line. The clump size was classified according to the number of fronds observed: (a) 1–3 fronds, (b) 4–9 fronds and (c) 10+ fronds. In July, August and September 2012–2014, three samples of *P. pavonica* clumps were collected from the sheltered and exposed sites to compare the size frequency and growth of the fronds. Collections (*n* = 100–200 fronds) were made from randomly selected smaller clumps of 10–20 fronds rather than larger clumps, as frond size in macroalgae can be density-dependent, yet thought to be less so in the Dictyotales (Riosmena-Rodŕiguez and Ortũno-Aguirre 2009). Within 24 h, clumps were washed to remove sand and the maximum frond length between base of the stipe and frond apex was measured with callipers to the nearest millimetre (Fig. 2). Populations were monitored in the early spring, autumn and winter months to establish the phenology of development and dieback.

### Tetrasporangial development and spore production

Fronds at sheltered and exposed populations were examined weekly during spring and summer to record the onset of tetrasporangial development and for the presence of gametangia. Tetrasporangia are located within concentric zones or rings of approximately 1 mm width on the upper surface of the frond, surrounded by a protective indusium, and are usually associated with bands of hairs (Carter 1927). Initial development of the tetraspores occurs within the outermost ring at the apical inrolled margin of the frond. Towards the base of the frond, sporangia are at a later stage of development and the third or fourth visible ring from the apex usually contains mature tetraspores. Tetraspores within each ring are at the same stage of development (Carter 1927). To determine the relationship between frond si*z*e and tetraspore production, several clumps were collected at random from the shore at Bembridge in July 2012. Fresh fronds were examined using stereo (40×) and high-power (200×) compound microscopy for the presence of oogonia, antheridia and tetrasporangia. To measure spore density within each ring, samples of the frond were removed using a scalpel blade and placed in a drop of seawater on a microscope slide with a cover slip. Using a micrometer eyepiece at 200×, five random (2 mm × 2 mm) ‘quadrats’ were positioned across the outermost sporangial ring containing mature tetraspores, usually visible ring 3 or 4 (ESM Fig.1), and the number of spores within each quadrat were counted. Viewed from the surface, tetraspores are rounded with diameter 50–60 µm (author unpubl). To estimate the number of tetraspores produced at any one point in time, the mean spore density (no. spores per mm^2^) was multiplied by width (1 mm) and the length of the tetrasporangial ring, which was measured with a piece of cotton. To calculate the potential number of spores produced by the frond up to the time of sampling, the lengths of all other visible inner (older) tetrasporangial rings were also measured and the number of spores that had been produced on these rings was estimated from the mean spore density calculated above. The relationship between potential spore production and frond length was determined using linear regression. Estimates of potential spore production per mean frond size were calculated for the months of July, August and September of 2012, 2013 and 2014.

To establish whether the rate of tetraspore production at particular sites differed among years 2009–2014, the minimum perpendicular width of all visible tetrasporangial rings on mature fronds in herbarium samples was recorded and measured with callipers to the nearest 0.1 mm. The number of tetraspores yet to be released and remaining on the inner (older) rings (4+) was also counted and compared with mean density in ring 3 to estimate the mean percentage spore release.

### Statistical analysis

Statistical analysis was conducted using ‘R’ (R Core Team 2013). Welch’s ‘*t*’ tests were used to determine the significance of differences in frond lengths and potential spore production between sheltered and exposed sites in each of three summer months during 2013 and 2014.

## Results

### Time-series analysis 1680–2014

**Fig. 3:**
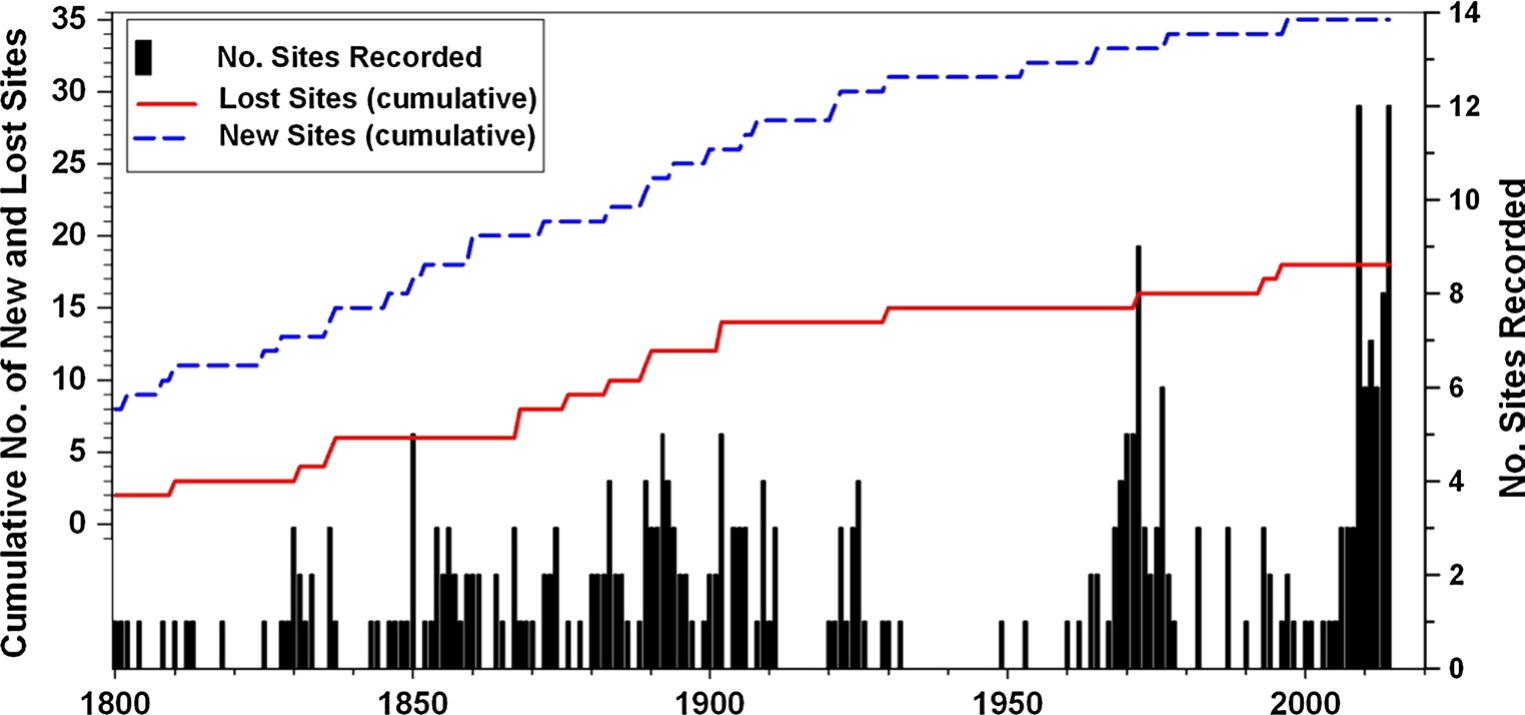
Annual number of recorded sites of *Padina pavonica* in southern England between 1800 and 2014, shown as *bars*. Peak periods of recording effort are the latter part of the nineteenth century, the 1960–1970s and most recently since 2000. Also shown is the date of first and last record at each location presented as the cumulative number of new and ‘lost’ sites. Seven sites were known prior to 1800, one of which has not been rediscovered

**Fig. 4:**
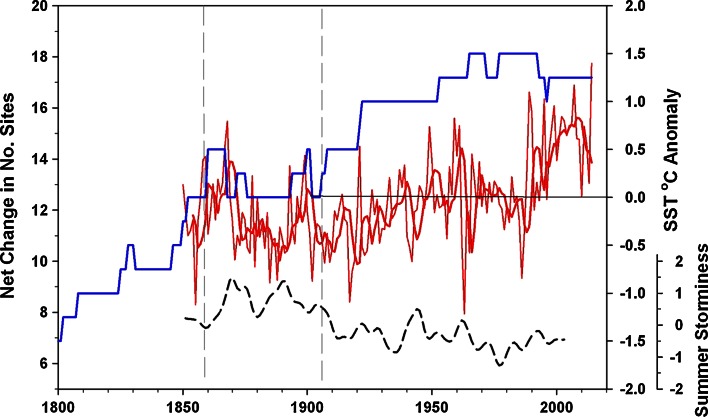
Net changes in number of sites with *P. pavonica* populations on the south coast of England (cumulative site gains minus cumulative site loss) between 1800 and 2014. Annual SST (and 5-year smoothed mean) anomalies (1960–1991 baseline) from 1850 to 1869 were extracted from the HadSST 3.1.1.0 data (Kennedy et al. 2011a, b) and from 1870 to 2014 extracted from the HadISST1 data set (Rayner et al. 2003). Summer storm index data from Cornes and Jones (2011). *Vertical dashed lines* indicate cooler and stormier period in mid-nineteenth century

The earliest record of *Padina pavonica* in England is from Exmouth in 1680 during the coldest period of the Little Ice Age (Table 2 site 30, Fig. 1) when mean temperatures in the Northern Hemisphere are estimated to have been 1 °C lower than present (Mann and Bradley 1999; Mann 2002). The longest continuous record of any extant population is from Nothe Rocks at Weymouth, where records go back to 1796. There have been three main phases of intensive recording activity: the latter half of the nineteenth century, the 1960–1970s and mid-2000s to present. Figure 3 shows the steady rise in the number of recorded sites in southern Britain since 1800 totalling 35 authenticated locations. Between 1680 and 1902, *P. pavonica* was recorded from 26 sites in southern Britain, yet over half of these sites had been ‘lost’ by the end of that period. Of these 14 ‘lost sites’, 8 disappeared over the period 1868–1902 (approximately 1 site every 4 years). *Padina* sites were ‘disappearing’ as rapidly as new localities were being found. The most frequently recorded site was Torbay in the west of the region where the species was recorded in 41 years since first being noted in Torquay in 1825. Over 70 % of these records, which included herbarium specimens, were prior to 1900, and in some of these years the species was recorded by more than a single observer. A single population (Boscastle) reappeared in 1993 having not been seen since first recorded in 1906, and a new site in Wales (Westdale Bay) was discovered in 1997. There has been no reappearance of *P. pavonica* at other historical sites. Between 1903 and 2000, an additional 4 sites were ‘lost’; therefore, 18 of the 35 sites known from 1680 to 2000 have been lost. The period in the late nineteenth century coincided with lower sea temperatures and higher frequency of summer storm events (Fig. 4). Sea temperatures have increased during the twentieth century and have risen sharply since the 1990s, whereas summer storm frequency has reduced over this period.

### Current distribution

Between 2000 and 2014, *Padina pavonica* was found at 17 of the 35 sites known in southern Britain. Since the last survey in the 1960s–1970s (Price et al. 1979), it was re-found at all except one of the sites (Lulworth Cove). Populations at five sites not recorded in the 1960–1970s (Luccombe, Compton Bay, Boscastle, Plymouth and Westdale Bay) have subsequently been reported; however, only populations at Luccombe, Compton Bay and Plymouth were located in the current survey. Except at Westdale Bay, *P. pavonica* has not been found at sites beyond previously recorded locations, despite intensive survey work by the authors and other field workers along the British coast.

*Padina pavonica* clumps were mostly found in rock pools between Mean High Water Neaps (MHWN) and Extreme Low Water Springs (ELWS), and habitat was comparable with historical accounts. Where annual surveys were possible (2009–2014), *P. pavonica* was found each year at all sites except at Lyme Regis where it was not recorded in 2011–2013 as rock pools were full of sediment. In 2012, the population at Ethel Point at Bembridge did not develop as all rock pools were smothered by 15–25 cm depth of sand. At Compton Bay, abundance varied between ‘Common’ in 2009 and ‘Rare’ in 2013 and 2014, when mid-shore pools appeared scoured due to large movements of sand during winter storms. Summer abundance varied within sites, often associated with sand movements. Sea urchins, which are known grazers on *Padina* spp. in the Mediterranean and elsewhere (Johnson et al. 2012), are rare in the intertidal zone on the central south coast of England and were not observed.

### Responses to sea temperature and disturbance

The years 2013 and 2014 were notable for contrasting mean spring sea temperatures (March–May). In 2013, spring SST was the lowest for 27 years and the fifth coolest for 50 years (mean 9.26 °C; anomaly −0.63 °C). Yet the spring of 2014 was the second warmest for 50 years (mean 11.52 °C; anomaly 1.62 °C). Compared to HadISST, inshore sea temperature data from Bramblemet weather buoy revealed similar trends, although spring values for 2013 and 2014 were significantly higher at 11.08 °C (HadISST +1.82 °C) and 13.98 °C (HadISST +2.46 °C), respectively (Solentmet Support Group 2015).

**Fig. 5:**
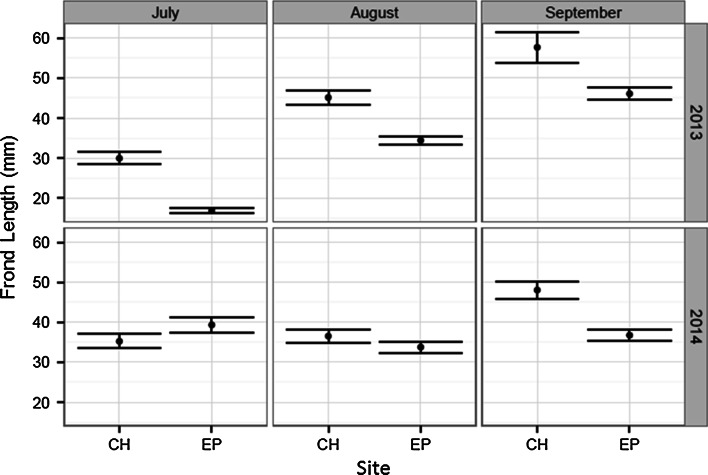
Mean frond length of populations at Colonels Hard (CH: sheltered) and Ethel Point (EP: exposed), Bembridge, Isle of Wight, in summer 2013 (cool spring) and 2014 (warm spring, August storms). The population at Ethel Point had been smothered by sand in 2012. *Error bars* show 95 % confidence intervals

During spring and summer of 2012, the exposed ledge at Ethel Point, Bembridge, was completely covered by 15–25 cm depth of sand and no *P. pavonica* or other seaweed was observed on the ledge during this period. However, the sheltered population at Colonels Hard developed normally and density was 24 clumps m^−2^ by August. In 2013 the appearance of *P. pavonica* at all sites along the south coast of England was delayed due to the cool spring. Growth at the sheltered site had commenced by June 15, yet although sand at the exposed shore had cleared by 26 May 2013, it was not until 21 July that fronds of *P. pavonica* were observed. The ledge remained relatively clear of sand for the remainder of the summer, and *P. pavonica* continued to grow over this period. Mean clump densities at the two sites in August 2013 were similar (13 clumps m^−2^ in the pool at Colonels Hard and 10 clumps m^−2^ at Ethel Point). By July, frond development at the exposed ledge was significantly retarded compared to the sheltered location (Fig. 5; Table 3). Mid-summer growth rates of both populations were almost identical, yet >10 mm difference in frond size between the populations was maintained until September. The spring of 2014 was exceptionally warm, and initial appearance of *P. pavonica* fronds at shores across the whole region was 14–30 days earlier than in 2013. Although sand at the exposed ledge at Ethel Point returned in the spring of 2014 (50 % coverage, max 5 cm depth), frond size in July was significantly higher compared to the sheltered site at Colonels Hard (Fig. 5; Table 3). Mid-summer growth between August and September was similar on both shores. However, due to storms in August, larger fronds were lost at both sites. By early September, *P. pavonica* at the exposed ledge at Ethel Point was ‘Rare’, yet at the sheltered site at Colonels Hard, *P. pavonica* remained ‘Abundant’ and tetrasporangial fronds were present until December.

**Table 4:** Mean frond length growth rates (mm day^−1^) at Bembridge, Isle of Wight, in 2013 and 2014. In 2012, the site at Ethel Point was buried by sand to May 2013

	15 June–21 July 2013	21 July–14 Sept 2013	24 May–10 July 2014	8 Aug–5 Sept 2014
Ethel Point	Absent	0.52 (*0.11*)	0.84 (*0.2*)	0.44 *(0.01*)
Colonels Hard	0.83 (*0.25*)	0.51 (*0.11*)	0.75 (*0.17*)	0.41 (*0.005*)

Values in parentheses show ±SD

**Fig. 6:**
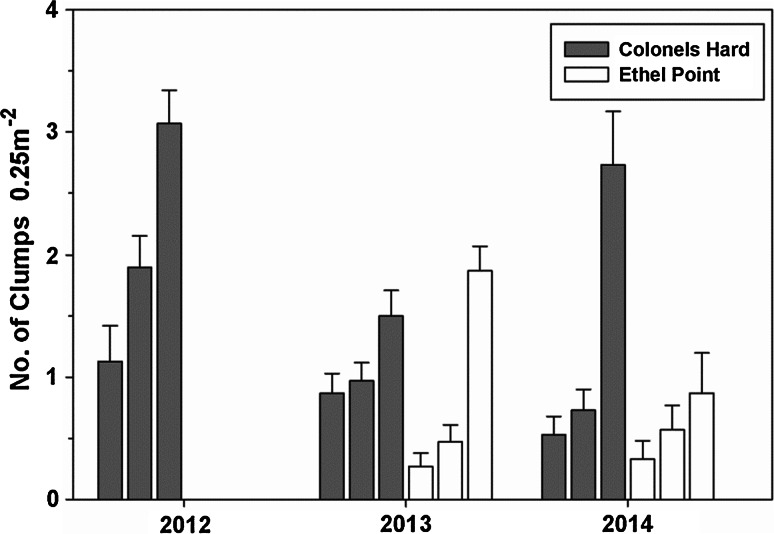
Mean density of small-, medium- and large-sized clumps of *Padina pavonica* at a sheltered location (Colonels Hard) and exposed ledge (Ethel Point) at Bembridge, Isle of Wight, south coast of England (0.25 m^2^ quadrats, *n* = 30). The three categories of clump size shown for each location are *left* to *right* 1–3, 4–9 and 10+ fronds. The ledge at Ethel Point was smothered by 15–20 cm of medium sand during 2012, and no *Padina* was observed.* Error bars* show +SE. See text for further details

Despite complete sand-smothering in 2012, vegetative rhizoids survived as large clumps of fronds appeared in both 2013 and 2014 (Fig. 6). Smaller clumps, possibly more recent recruits, were evident in both populations, although at higher density at the sheltered location.

No gametangial development was observed in over 2000 fronds examined during the survey period at sites across the region. Tetraspores were usually visible from June, and at some sheltered locations, rings with mature spores could still be seen in December. Large, mature fronds of 50 mm length could have eight or more visible tetrasporangial rings. Normally, at 40× magnification, only the outer three visible rings have developing or mature sporangia (ESM Fig.1). From estimates of early frond growth (May–July) of ~0.8 mm day^−1^ (Table 4), it would take 25 days for fronds to reach 20 mm in length, the minimum frond size for which tetrasporangial rings have been observed. Assuming length of maturity of 20 mm, a frond of age 63 days and length 50 mm may therefore have produced between 4 and 8 generations of tetraspores. Growth rate from July–September is lower at ~0.4 mm day^−1^; therefore, an additional 2–4 generations of tetraspores might be expected to be produced over this period.

Tetrasporangial rings are approximately 1 mm width, and spores within each ring appear to develop synchronously. Maximum spore densities were 55 mm^−2^ (*x̅* ± SE = 25 ± 1.8 mm^−2^).

The mean spore density remaining on the tetrasporangial ring immediately inside that containing the mature spores (usually ring 4) was 17 mm^−2^, suggesting that ~70 % are released over a short time period of perhaps a few days. Few spores were found on any of the older rings.

**Fig. 7:**
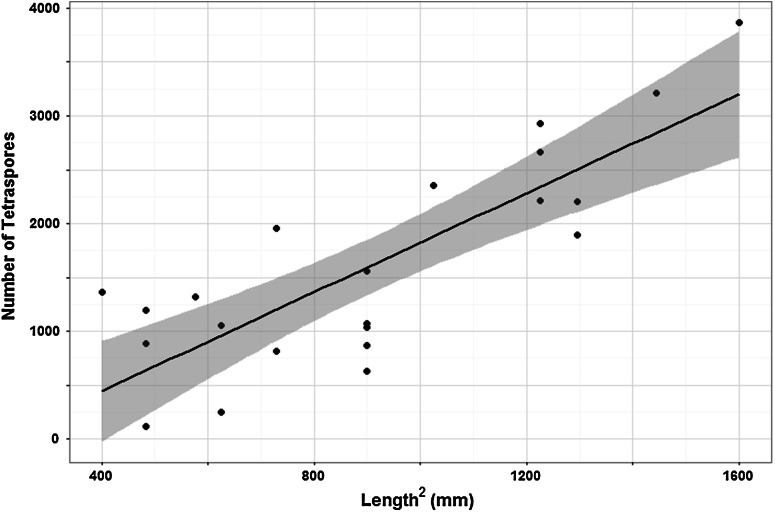
Relationship between *P. pavonica* frond length-squared and estimated production of number of tetraspores for Bembridge: *R* ^2^ = 0.65; *y* = 2.298*x* − 474.74. *Shaded area* denotes 95 % confidence intervals

**Fig. 8:**
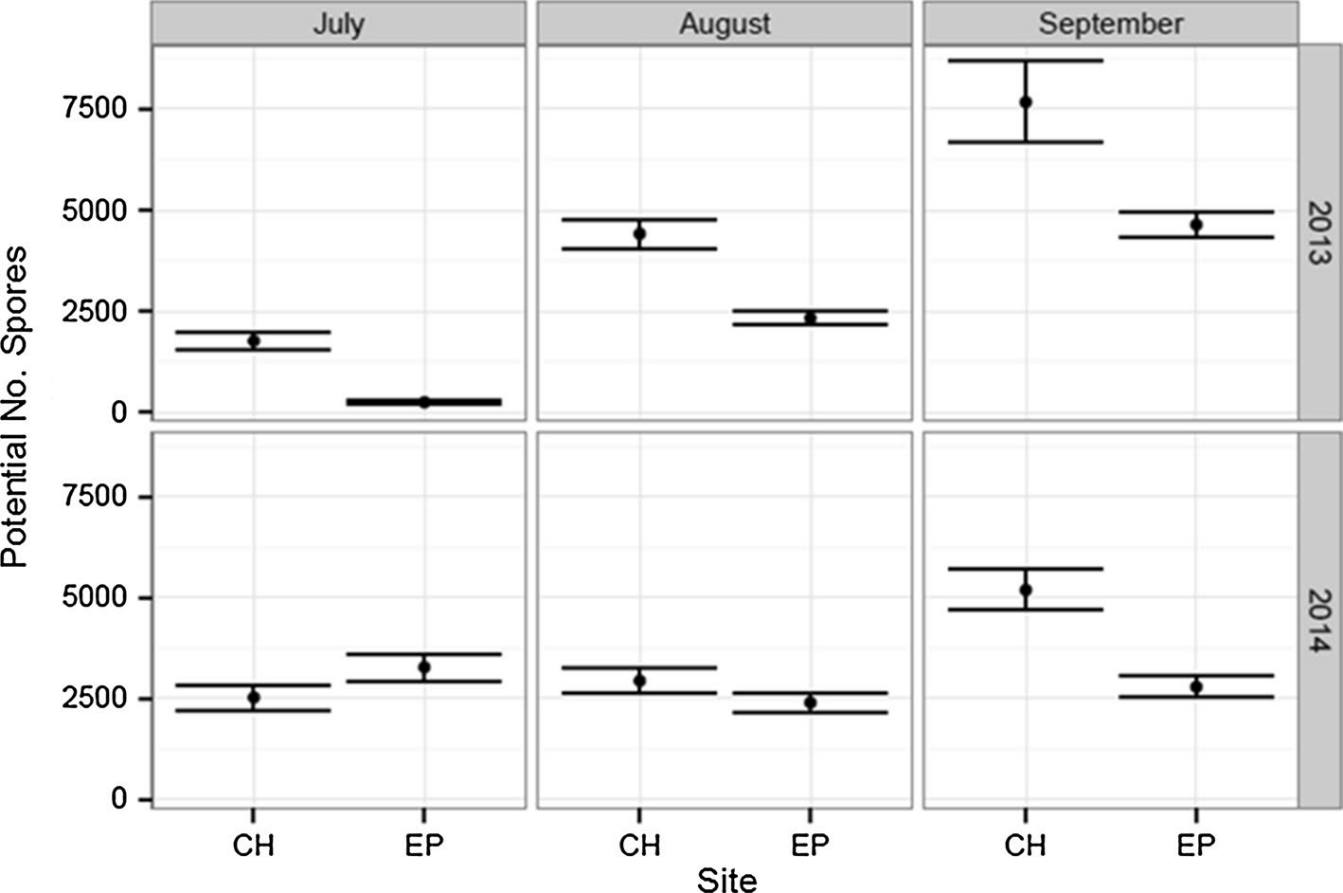
Potential mean tetraspore production per mean frond length in populations at sheltered (*CH* Colonels Hard) and exposed (*EP* Ethel Point) sites at Bembridge, Isle of Wight, in 2013 (cool spring) and 2014 (warm spring, August storms). *Error bars* show 95 % confidence intervals. Population at Ethel Point was smothered by sand in 2012: sand had cleared by May 2013. Spore production based on regression slopes in Fig. 5

From linear regression analysis, frond length-squared was found to be the best predictor of potential number of spores (*R*^2^ = 0.65; Fig. 7). Potential spore production increases between July and August, associated with a general increase in frond length (Fig. 8; Table 5). At Bembridge, this was evident at the sheltered location in all years and at the exposed ledge in 2013. In 2012, the exposed ledge was completely smothered by sand for the entire summer and fronds failed to appear and no spores were produced. In 2013 and 2014, fronds at the sheltered site were mostly larger and had significantly greater spore production than clumps at the exposed site; here, the population can fail to retain many large fronds due to water movements and physical damage and abrasion from shifting sands. Given calm conditions, early growth at both sites is similar and was slightly greater at the exposed ledge in July 2014. In the cooler years of 2012 and 2013, potential tetraspore production by July was less due to retarded growth and later appearance of fronds. Yet despite later development, combining data from both sheltered and exposed sites, the mean potential production of tetraspores in both August and September was significantly greater than in the warmer year of 2014 (Table 6); fronds that had grown rapidly in the warm spring had become weak and succumbed to storms in mid-August. By mid-September clumps, at the sheltered location had grown and produced new and larger fronds and had a greater number of tetraspores compared to the exposed ledge.

**Table 5:** Welch’s ‘*t*’ test results indicating significance of differences between mean values of potential spore production at sheltered (*CH* Colonels Hard) and exposed (*EP* Ethel Point) sites at Bembridge, measured during summer months in 2013 and 2014

Year	Month	Mean no. spores CH	Mean no. spores EP	*t*	*df*	*p*
2013	July	1765	247	13.74	146.28	<0.001
	August	4416	2327	10.37	169.56	<0.001
	September	7674	4640	5.78	72.70	<0.001
2014	July	2532	3280	3.25	172.19	0.001
	August	2944	2404	2.66	382.71	0.008
	September	5190	2791	8.42	195.82	<0.001

**Table 6:** Welch’s ‘*t*’ test results indicating significance of differences between mean values of potential spore production at Bembridge in summer 2013 and 2014, combining data from both sheltered (*CH* Colonels Hard) and exposed (*EP* Ethel Point) sites

Month	Mean no. spores 2013	Mean no. spores 2014	*t*	*df*	*p*
August	3374	2669	4.36	653	<0.001
September	5405	4024	5.53	497	<0.001

## Discussion

On the south coast of England, *P. pavonica* is known to have been present for at least 330 years. To the best of our knowledge, the records in Price et al. (1979) combined with recent observations over the past thirty years are the longest compilation and review for any marine algal species. Some extant populations are known to have existed for over 200 years, such as at Weymouth and Lyme Regis. This record is remarkable and unusual for sessile species and particularly so for marine algae. The precision of geographical location was previously not as accurate as it is now; however, several populations can be traced to surviving shore localities. Over this recorded period, populations persisted during the coolest phase of the Little Ice Age at the end of the seventeenth century (Mann 2002), notably cold winters of 1947, 1963 (Crisp 1964), 2010–2011 and severe storms (Lamb 1991).

There had been five site ‘extinctions’ prior to 1868 from the east, central and west of the region. Yet in the mid-late nineteenth century, there appears to have been a perturbation in the continuity of known sites. The discontinuity of recording at a site does not necessarily indicate extinction, even for a relatively well recorded species such as *P. pavonica,* as it is possible that the species has occasionally reappeared, perhaps from inconspicuous vegetative rhizoids, or been overlooked. It is therefore impossible to be precise about the dates of site extinction as we only have evidence of the last reported observation. It is most likely that the number of recorded populations increased in the eighteenth and early nineteenth centuries due to a rising number of collectors attracted to the species distinctive morphology. We cannot discount the possibility that some of these populations were sufficiently small to have been vulnerable to overcollecting and were depleted over several years; such was the interest in the activity during that period. Making herbarium collections of seaweeds was a fashionable Victorian pastime for the gentry (at least 100 specimens of *P. pavonica* have been deposited in herbaria within British Isles (Seaweed Collections Online 2015). ‘Extinct’ sites do not share any common characteristics except that several are in close proximity, suggesting local factors could be important; Margate and Dover on the North Sea coast (Fig. 1) are exposed to colder winters and spring temperatures, whereas Teignmouth, Dawlish and Exmouth are located at the mouths of estuaries notable for shifting sands. Swanage and Studland are in the region of least tidal range and smallest available habitat. Like so many other sites in the region, the population at Steephill Cove would have been susceptible to smothering by landslip and coastal erosion. As overseas trade increased during the nineteenth century, port and harbour developments were prolific and populations may have been affected by localised changes in wave exposure, currents and sediment transport. There is no longitudinal or latitudinal gradient in site extinctions over the long term. However, there is evidence that summer seasons during the latter half of the nineteenth century were characterised by relatively unsettled and stormy conditions (Lamb 1991; Wang et al. 2009; Cornes and Jones 2011). Folland et al. (2009) show the existence of a high degree of spatial coherence in storm activity across the North Sea, British Isles and central-west Europe during the high-summer months (July and August) in connection with the leading mode of atmospheric circulation variability during that time of the year: the Summer North Atlantic Oscillation (SNAO). Their analysis also demonstrates a strengthening of storm activity across that region during the late nineteenth century compared to the relative quiescence of the post-1960 period. Further evidence for the unsettled climate during this period is shown by the SST times-series (Fig. 4), which indicates sustained lower temperatures over this period (Rayner et al. 2003). Such weather patterns are also associated with variable cloud cover and lower mean solar irradiance. During the twentieth century, populations may have benefitted from the combined effects of warming and more settled summer conditions. Elevated CO_2_ and nutrients levels may have enhanced physiological performance, as demonstrated experimentally for *P. pavonica* in the Mediterranean (Celis-Plá et al. 2015).

### Sensitivity to temperature and disturbance

The hypotheses tested in relation to frond growth and production of tetraspores is mostly supported by the short-term experiments and observations. The production of tetraspores was higher in the warmer spring and growth and spore production was generally less at the exposed site, as larger fronds bearing larger numbers of tetraspores are more vulnerable to summer storm damage and sloughed off. High wave exposure is thought to be responsible for smaller plants and lower recruitment of *P.boryana* Thivy in the Indian Ocean due to physical destruction of thalli (Wichachucherd et al. 2010). The extent to which the cool spring, in combination with sand-smothering, contributed to the delayed appearance and growth of the exposed population at Ethel Point in 2013 is unknown. In the mid-nineteenth century, lower temperatures and high storm frequency and/or intensity may have caused increased stress and disturbance of populations above thresholds required for population survival. Yet the hypothesis that unfavourable weather conditions caused regional decline of *P. pavonica* is challenged by references to its abundance at certain sites during or shortly after that period (Table 2; Price et al. 1979). Notwithstanding regional adaptation, the sensitivity of individual populations is likely to be determined by exposure to local stress and disturbances. Physiological adaptation and the presence of local ecotypes could be very important in seaweeds (Breeman 1988; Harley et al. 2012; Ferreira et al. 2014), and there has been a suggestion of local adaptation of *P. pavonica* to nutrient enrichment (Price et al. 1979, p 17).

The species presence during the Little Ice Age, and prevalence in the late 1960s and 1970s following the severe cold winter of 1962–1963, when many other marine organisms perished (Crisp 1964), indicates a tolerance of the rhizoidal stage to extremely low winter temperatures. Our data show that frond growth and the duration of spore production are sensitive to sea temperature. A longer period of spore production with multiple releases should theoretically increase the probability of dispersal into a favourable environment for germination, recruitment and population persistence. Where populations are relatively protected from storms, clump survival is better and the duration of spore production is long. Yet our study shows that earlier growth in warmer springs can make fronds more susceptible to storm damage, which can limit potential spore production in autumn (Fig. 8). Projected increases in SST combined with extreme storm events (IPCC 2014) could therefore have antagonistic effects on spore production that may impact on recruitment and population size; warmer spring temperatures can accelerate spore production, whereas summer storm damage will reduce spore production. It has been observed that summer storms and sand movements affect clump abundance, smothering and frond-size frequency at other sites in the region. However, the spatial extent of disturbance has been variable between sites and years (author’s observations). In experiments on subtidal reef patches in the Mediterranean, *P. pavonica* recruitment and survival were dependant on the intensity, area and timing of disturbance from sand deposition (Airoldi 1998, 2000). In this study, the ‘recovery’ of the exposed population from sand-smothering may have been due to the survival of perennating rhizoids and/or spores within the sediment or clay substratum. A longer period of sand-smothering or complete erosion of the substrate surface may negatively affect survival. Sheltered habitats on these shores, such as deeper rock pools, probably provide refugia and contribute to population persistence.

Separating sensitivity to temperature and solar radiation is particularly difficult as they often co-vary. We cannot be certain which of these variables is most important as different stages of a species life history may have different tolerance thresholds (Breeman 1988). Light irradiance is a major factor influencing macroalgal shore height occupation (Gomez et al. 2004; Rohde et al. 2008). Considering the species higher abundance in southern latitudes and greater depth penetration in the Mediterranean, light levels may be particularly significant for growth (Price et al. 1979). In culture experiments, Jones (1999) found that light levels were more important to early growth of *P. pavonica* than water temperature. It is therefore possible that population decline in the late nineteenth century was the result of a combination of stressors including lower irradiance due to longer periods of cloud cover, with the common factor being the state of the SNAO.

Although there have been localised extinctions of populations of *P. pavonica*, multiple spore releases, together with vegetative perennation and possibly propagation, must have contributed significantly to the species persistence and survival in the region over the past 330 years. During cooler and stormier periods, occasional years with warmer and longer growing conditions may have favoured spore production, recruitment and enabled persistence. The fate of dislodged vegetative fragments and their role in local propagation and recruitment are unknown. It is possible that the dispersal of vegetative propagules, perhaps caused by frond damage and fragmentation due to storm events, could be important to local recruitment. The presence of large clumps in pools that had been smothered during the previous year could indicate tolerance of the perennating rhizoidal network and its importance in sustaining populations during short-term yet intense physical disturbances. We know from accurate dGPS mapping (author unpubl) that many clumps appear in exactly the same pools each year, suggesting that perennation, vegetative propagation and local spore germination might currently be occurring, separately or in combination. The species’ resilience to periods of sand-smothering and abrasion may have facilitated survival on dynamic shores consisting of softer substrata where other, potentially competitive, algal species would fail. It is argued that disturbance events may be particularly important at the periphery of species ranges in regulating interspecific interactions (Araujo et al. 2012). High regional and local variation in topography, particularly the presence of sheltered pools, must also be beneficial.

### Potential for range extension

Due to rising sea and air temperatures, it has been suggested that range extension of *P. pavonica* at its northern periphery in the British Isles is now possible (Hiscock et al. 2004). The species is currently common or abundant at ten sites along the south coast of England (Fig. 1), and several species of warm-temperate intertidal macrofauna and flora have undergone range extensions on rocky shores in the region over the past fifteen years (Herbert et al. 2003; Mieszkowska et al. 2006; Hawkins et al. 2009). Although there are a few pre-twentieth century records of *P. pavonica* in northern Britain, based on knowledge of preferred habitat and known environmental tolerances, these were considered doubtful or had questionable authenticity by Price et al. (1979). However, a record from Ayr on the west coast of Scotland in the mid-nineteenth century was, with considerable reservation, accepted as a rare occurrence and currently constitutes the known northern limit of the species. Yet although sea temperatures are now significantly warmer compared to the earlier surveys in the cooler 1960s and 1970s, there has been no substantial change in distribution in Britain over the past 35 years. It is unknown whether the species’ occasional appearance at localities that are distant from core populations (e.g. Table 2 site 32, Boscastle) is due to long-distance propagule dispersal, a regeneration following a period of dormancy or infrequent recording. Reproductive connectivity between populations is unknown. However, there is a growing consensus that for a wide variety of taxa, local replenishment of populations is the norm, with most propagules being dispersed into and recruiting from ‘local waters’ (Shanks et al. 2003). Coleman et al. (2011) found low-population connectivity among intertidal algae, and hydrographic barriers to larval dispersal have been demonstrated in the study region (Herbert et al. 2009; Keith et al. 2011). The low profile of *P. pavonica* is also likely to limit spore dispersal (Gaylord et al. 2002), and it is most probable that the relatively heavy tetraspores sink to the bottom of parent rock pools. Yet although spore dispersal is thought to be limited, model algal spores are sensitive to strong, short-duration turbulent transport events and the spread or invasion rate of macroalgae could potentially be 10–100 kilometres per year (Kinlan and Gaines 2003; Kinlan et al. 2005). On the Portuguese coast, a 187-km-range extension of *P. pavonica* since the 1950–1960s has been attributed to recent warming (Lima et al.^2007^).

## Conclusions

The long historic record on the south coast of England is evidence of population tenacity and survival over three centuries of variable weather. Periods of rapid environmental change can be damaging to populations if species fail to acclimatise or adapt to change (O’Conner et al. 2012). Yet where there is sufficiently sheltered habitat and rock pool refugia, *P. pavonica* may fare better than many species due to resilient vegetative stages that have enabled persistence during cooler and stormier periods and multiple releases of tetraspores during calmer and warmer summers. Antagonistic responses to multiple stressors and disturbances make future predictions of survival and distribution difficult. Coastal management interventions as a result of sea-level rise and increased storminess may cause local variation in sediment transport and wave exposure that increase population susceptibility to physical damage. To improve our ability to predict impacts of environmental change, stress and disturbances, it is vital that systematic surveys are continued as with canopy-forming seaweed species (Yesson et al. 2015). Comparative investigations into the morphology and genome of northern and southern populations of *P. pavonica* may reveal regional adaptations.

## Electronic supplementary material

Below is the link to the electronic supplementary material.

Supplementary material 1 (PDF 203 kb)

## References

[CR1] Airoldi L (1998). Roles of disturbance, sediment stress and substratum retention on spatial dominance in algal turf. Ecology.

[CR2] Airoldi L (2000). Responses of algae with different life histories to temporal and spatial of variability of disturbance in subtidal reefs. Mar Ecol Prog Ser.

[CR3] Airoldi L (2003). The effects of sedimentation on rocky coast assemblages. Oceanogr Mar Biol Ann Rev.

[CR4] Araujo R, Arenas F, Åberg P, Sousa-Pinto I, Serrão EA (2012). The role of disturbance in differential regulation of co-occurring brown algae species: interactive effects of sediment deposition, abrasion and grazing on algae recruits. J Exp Mar Biol Ecol.

[CR5] Betancor S, Tuya F, Gil-Díaz T, Figueroa FL, Haroun R (2014). Effects of a submarine eruption on the performance of two brown seaweeds. J Sea Res.

[CR6] Breeman AM (1988). Relative importance of temperature and other factors in determining geographic boundaries of seaweeds: experimental and phonological evidence. Helogolander Meersun.

[CR7] Carter PW (1927). The life history of *Padina pavonia* I. The structure and cytology of the tetrasporangial plant. Ann Bot.

[CR8] Celis-Plá PSM, Hall-Spencer JM, Horta PA, Milazzo M, Korbee N, Cornwall CE, Figueroa FL (2015). Macroalgal responses to ocean acidification depend on nutrient and light levels. Front Mar Sci.

[CR9] Coleman MA, Chambers J, Knott NA, Malcolm HA, Harasti D, Jordan A, Kelaher BP (2011). Connectivity within and among a network of temperate marine reserves. PLoS ONE.

[CR10] Cornes RC, Jones PD (2011). An examination of storm activity in the northeast Atlantic region over the 1851–2003 period using the EMULATE gridded MSLP data series. J Geophys Res-Atmos.

[CR11] Crisp DJ (ed) (1964) The effects of the severe winter of 1962–1963 on marine life in Britain. J Anim Ecol 33:165–210

[CR12] Crisp DJ, Southward AJ (1958). The distribution of intertidal organisms along the coasts of the English Channel. J Mar Biol Ass UK.

[CR13] Dixon PS (1965). Perennation, vegetative propagation and algal life histories, with special reference to *Asparagopsis* and other Rhodophyta. Botanica Gothoburg.

[CR14] Eggert A, Wiencke C, Bischof K (2012). Seaweed responses to temperature. Seaweed Biology.

[CR15] Eriksson BK, Johansson G (2005). Effects of sedimentation on macroalgae: species specific responses are related to reproductive traits. Oecologia.

[CR16] Ferreira JG, Arenas F, Martınez B, Hawkins SJ, Jenkins SR (2014). Physiological response of fucoid algae to environmental stress: comparing range centre and southern populations. New Phytol.

[CR17] Folland CK, Knight J, Linderholm HW, Fereday D, Ineson S, Hurrell JW (2009). The Summer North Atlantic Oscillation: past, present, and future. J Clim.

[CR18] Forbes E (1858). The distribution of marine life, illustrated chiefly by fishes, molluscs and radiate. Johnston AK (undated) Physical atlas.

[CR19] Gaylord B, Reed DC, Raimondi PT, Washburn L, McLean SR (2002). A physically based model of macroalgal spore dispersal in the wave and current dominated nearshore. Ecology.

[CR20] Gil-Díaz T, Haroun R, Tuya F, Betancor B, Viera-Rodríguez MA (2014). Effects of ocean acidification on the brown alga *Padina pavonica*: decalcification due to acute and chronic events. PLoS ONE.

[CR21] Gomez I, Lopez-Figueroa F, Ulloa N, Morales V, Lovengreen C, Huovinen P, Hess S (2004). Patterns of photosynthesis in 18 species of intertidal macroalgae from southern Chile. Mar Ecol-Prog Ser.

[CR22] Gómez-Garreta A, Rull-Lluch J, Barceló Marti´ MC, Ribera Siguan MA (2007). On the presence of fertile gametophytes of *Padina pavonica* (Dictyotales, Phaeophyceae) from the Iberian coasts. Anales Jard. Bot Madrid.

[CR23] Grime JP (1977). Evidence for the existence of three primary strategies in plants and its relevance to ecological and evolutionary theory. Am Nat.

[CR24] Guiry MD, Guiry G (2014). *AlgaeBase*. World-wide electronic publication, National University of Ireland, Galway. http://www.algaebase.org. Accessed 28 Nov 2015

[CR25] Harley CDG, Hughes AR, Hultgren KM, Miner BG, Sorte CJB, Thornber CS, Rodriguez LF, Tomanek L, Williams SL (2006). The impacts of climate change in coastal marine systems. Ecol Lett.

[CR26] Harley CDG, Anderson KM, Demes KW, Jorve JP, Kordas RL, Coyle TA (2012). Effects of climate change on global seaweed communities. J Phycol.

[CR27] Hawkins SJ, Sugden HE, Mieszkowska N, Moore P, Poloczanska E, Leaper R, Herbert RJH, Genner MJ, Moschella PS, Thompson RC, Jenkins SR, Southward AJ, Burrows MT (2009). Consequences of climate driven biodiversity changes for ecosystem functioning of North European Rocky Shores. Mar Ecol Prog Ser.

[CR28] Hawkins SJ, Firth LB, McHugh M, Poloczanska ES, Herbert RJH, Burrows MT, Kendall MA, Moore PJ, Thompson RC, Jenkins SR, Sims DW, Genner MJ, Mieszkowska N (2013). Data rescue and re-use: recycling old information to address new policy concerns. Mar Pol.

[CR29] Herbert RJH, Hawkins SJ, Sheader M, Southward AJ (2003). Range extension and reproduction of the barnacle *Balanus perforatus* in the eastern English Channel. J Mar Biol Assoc UK.

[CR30] Herbert RJH, Southward AJ, Clarke RT, Sheader M, Hawkins SJ (2009). Persistent border: an analysis of the geographic boundary of an intertidal species. Mar Ecol Prog Ser.

[CR31] Hiscock K (ed) (1998) Marine Nature Conservation Review. Benthic marine ecosystems: A Review of Current Knowledge for Great Britain and the North-east Atlantic, Joint Nature Conservation Committee, Peterborough

[CR32] Hiscock K, Southward A, Tittley I, Hawkins SJ (2004). Effects of changing temperature on benthic marine life in Britain and Ireland. Aquat Conserv.

[CR33] IPCC (2014) Climate change 2014: impacts, adaptation, and vulnerability. Part A: global and sectoral aspects. Contribution of Working Group II to the Fifth Assessment Report of the Intergovernmental Panel on Climate Change. In: Field, CB, Barros VR, Dokken DJ, Mach KJ, Mastrandrea MD, Bilir TE, Chatterjee M, Ebi KL, Estrada YO, Genova RC, Girma B, Kissel ES, Levy AN, MacCracken S, Mastrandrea PR, L.L. LL (eds) Cambridge University Press, Cambridge

[CR34] Jenkins SR, Hawkins SJ, Norton TA (1999). Direct and indirect effects of a macroalgal canopy and limpet grazing in structuring a sheltered inter-tidal community. Mar Ecol Prog Ser.

[CR35] Jenkins SR, Norton TA, Hawkins SJ (2004). Long term effects of *Ascophyllum nodosum* canopy removal on mid shore community structure. J Mar Biol Assoc UK.

[CR36] Johnson VR, Russell BD, Fabricius KE, Brownlee C, Hall-Spencer JM (2012). Temperate and tropical brown macroalgae thrive, despite decalcification, along natural CO_2_ gradients. Glob Change Biol.

[CR37] Jones LA (1999) Studies on littoral algae of the Isle of Wight and Solent region. PhD Thesis, University of Portsmouth, Portsmouth (UK)

[CR38] Keith SA, Herbert RJH, Norton PA, Hawkins SJ, Newton AC (2011). Individualistic species limitations of climate-induced expansions generated by meso-scale dispersal barriers. Divers Dist.

[CR39] Kennedy JJ, Rayner NA, Smith RO, Saunby M, Parker DE (2011). Reassessing biases and other uncertainties in sea-surface temperature observations since 1850 part 1: measurement and sampling errors. J Geophys Res.

[CR40] Kennedy JJ, Rayner NA, Smith RO, Saunby M, Parker DE (2011). Reassessing biases and other uncertainties in sea-surface temperature observations since 1850 part 2: biases and homogenisation. J Geophys Res.

[CR41] Kinlan BP, Gaines SD (2003). Propagule dispersal in marine and terrestrial environments: a community perspective. Ecology.

[CR42] Kinlan BP, Gaines SD, Lester SE (2005). Propagule dispersal and scales of marine community process. Divers Dist.

[CR43] Kraufvelin P, Salovius S (2004). Animal diversity in Baltic rock shore macroalgae: Can *Cladophora glomerata* compensate for lost *Fucus vesiculosus*?. Estuar Coast Shelf Sci.

[CR44] Lamb H (1991). Historic storms of the North Sea.

[CR45] Lima FP, Ribeiro P, Aqueiroz N, Hawkins SJ, Santos AM (2007). Do distributional shifts of northern and southern species of algae match the warming pattern?. Glob Change Biol.

[CR46] Mann ME, MacCracken MC, Perry JS (2002). The Earth system: physical and chemical dimensions of global environmental change: Little Ice Age. Encyclopedia of Global Environmental Change.

[CR47] Mann ME, Bradley RS (1999). Northern Hemisphere temperatures during the past Millennium: inferences, uncertainties, and limitations. Geophy Res Lett.

[CR48] Mieszkowska N, Kendall MA, Hawkins SJ, Leaper R, Williamson P, Hardman-Mountford NJ, Southward AJ (2006). Changes in the range of some common rocky shore species in Britain—A response to climate change?. Hydrobiologia.

[CR49] Mieszkowska N, Sugden H, Firth LB, Hawkins SJ (2014). The role of sustained observations in tracking impacts of environmental change on marine biodiversity and ecosystems. Philos T R Soc A.

[CR50] Milazzo M, Badalamenti F, Riggio S, Chemello R (2004). Patterns of algal recovery and small-scale effects of canopy removal as a result of human trampling on a Mediterranean rocky shallow community. Biol Conserv.

[CR51] NBN (2014) National biodiversity network gateway. https://data.nbn.org.uk/. Accessed 23 July 2015

[CR52] O’Conner MI, Selig ER, Pinsky ML, Altermatt F (2012). Toward a conceptual synthesis for climate change responses. Global Ecol Biogeogr.

[CR53] Padilla-Gaminõ JL, Carpenter RC (2007). Seasonal acclimatization of *Asparagopsis taxiformis* (Rhodophyta) from different biogeographic regions. Limnol Oceanogr.

[CR54] Price JH, Tittley I, Richardson WD (1979) The distribution of *Padina pavonica* (L.) Lamour. (Phaeophyta: Dictyotales) on British and adjacent European shores. Brit Mus (Natural History). Bot Ser 7:1–67

[CR63] R Core Team (2013) R: A language and environment for statistical computing. R Foundation for Statistical Computing, Vienna, Austria. http://www.R-project.org/

[CR55] Rayner NA, Parker DE, Horton EB, Folland CK, Alexander LV, Rowell DP, Kent EC, Kaplan A (2003). Global analyses of sea surface temperature, sea ice, and night marine air temperature since the late nineteenth century. J Geophys Res.

[CR56] Riosmena-Rodŕiguez R, Ortũno-Aguirre C (2009). Population structure and reproduction of *Padina concrescens* Thivy in southwest Baja California Peninsula, Mexico. Algae.

[CR57] Rohde S, Hiebenthal C, Wahl M, Karez R, Bischof K (2008). Decreased depth distribution of Fucus vesiculosus (Phaeophyceae) in the Western Baltic: effects of light deficiency and epibionts on growth and photosynthesis. EU J Phycol.

[CR59] Seaweed Collections Online (2015) http://seaweeds.myspecies.info/. Accessed 10 Aug 2015

[CR60] Shanks AL, Grantham BA, Carr MH (2003). Propagule dispersal distance and the size and spacing of marine reserves. Ecol Appl.

[CR61] Solentmet Support Group (2015) Bramblemet weather buoy. http://www.bramblemet.co.uk/(S(csyow4454nh12245in4uifiz))/default.aspx. Accessed 2 Nov 2015

[CR64] Umar MJ, McCook LJ, Price IR (1998). Effects of sediment deposition on the seaweed *Sargassum* on a fringing coral reef. Coral Reefs.

[CR65] Vaselli S, Bertocci I, Maggi E, Benedetti-Cecchi L (2008). Effects of mean intensity and temporal variance of sediment scouring events on assemblages of rocky shores. Mar Ecol Prog Ser.

[CR66] Wang XL, Zwiers FW, Swail VR, Feng Y (2009). Trends and variability of storminess in the northeast Atlantic region, 1874–2007. Clim Dyn.

[CR68] Wernberg T, Smale DA, Thomsen MS (2012). A decade of climate change experiments on marine organisms: procedures, patterns and problems. Glob Change Biol.

[CR69] Wichachucherd B, Liddle LB, Prathep A (2010). Population structure, recruitment, and succession of the brown alga, *Padina boryana* Thivy (Dictyotales, Heterokontophyta), at an exposed shore of Sirinart National Park and a sheltered area of Tang Khen Bay, Phuket Province, Thailand. Aquat Bot.

[CR70] Yesson C, Bush LB, Davies AJ, Maggs CA, Brodie J (2015). Large brown seaweeds of the British Isles: evidence of changes in abundance over four decades. Estuar Coast Shelf Sci.

