# Supplementary material for: The calcareous brown alga Padina pavonica in southern Britain: population change and tenacity over 300 years
Source: Mar Biol. 2016 Feb 10;163:46. doi: 10.1007/s00227-015-2805-7 (PMC4749647; doi:10.1007/s00227-015-2805-7)
Supplement: Supplementary file 1 — Supplementary material 1 (PDF 203 kb) [file 227_2015_2805_MOESM1_ESM.pdf]

The calcareous brown alga *Padina pavonica* in southern Britain:

Population change and tenacity over 300 years

Roger J.H. Herbert[1], Lisha Ma[1], Anne Marston[2], William F Farnham[3], Ian Tittley[4],

Richard C Cornes[5]

[1]Faculty of Science & Technology, Department of Life and Environmental Sciences, Bournemouth University,

Talbot Campus, Fern Barrow, Poole, Dorset, UK, BH12 5BB.

[2] Isle of Wight Local Records Centre, Seaclose Offices, Fairlee Road, Newport, Isle of Wight, UK, PO30 2QS.

[3] Portsmouth University.

[4] The Natural History Museum, Cromwell Road, London, UK SW7 5BD.

[5] Climatic Research Unit, School of Environmental Sciences, University of East Anglia,

E-mail: Corresponding author rherbert@bournemouth.ac.uk; Tel +44 (0) 1202965908

Electronic Supplementary Information

ESM Table 1. Locations within sites where records have been combined. These are mostly multiple localities referred to in Price et al. (1979) that are in close proximity, where place names have been updated or specific areas that have been included within the current survey.

County	Site (Table2)	Locations	Notes
Kent	Margate	Margate<br>Foreness Point	
Sussex	Bognor	Bognor<br>Bognor Rocks<br>Elmer<br>Littlehampton	This site has changed considerably since the 18th Century. During the current survey new rock sea defences at Elmer and a cobble shore at Littlehampton were also checked.
Isle of Wight	Bembridge	Bembridge<br>Bembridge Lagoon Bembridge Forelands,<br>Bembridge Lifeboat Station<br>Colonels Hard	
Isle of Wight	Shanklin	Shanklin<br>Shanklin Horse Ledge<br>Sandown<br>Lake<br>Sandown Bay	A herbarium record for Shanklin date June 1872 was obtained from National Museum Wales.
Isle of Wight	Luccombe	Shanklin Luccombe Ledge<br>Yellow Ledge	Price et al (1979) considered the record from Luccombe Ledge to be from Horse Ledge. However recent records from Yellow Ledge, which is closer to Luccombe, suggest that this is as likely to be the primary site and is considered so in this paper.
Isle of Wight	Steephill	Steephill<br>Steephill Cove	Two undated 19th Century herbarium specimens from Steephil Cove specimens were located in National Museum Wales but were not included.
Isle of Wight	Compton Bay	Brook Bay, Compton Bay<br>Hanover Point	
Isle of Wight	Colwell Bay	Colwell Bay<br>Colwell Bay (Warden Point)<br>How Ledge	
Dorset	Kimmeridge	Kimmeridge<br>Gad Cliff, 200m east of Wagon Rock (Brandy Bay)<br>Broad Bench, Charnel, The Flats	

County	Site (Table2)	Locations	Notes
	Osmington	Osmington Mills<br>Osmington	Osmington is 1 Km west of Osmington Mills
	Weymouth	Weymouth<br>Nothe Rocks<br>Newtons Cove	
	Portland Harbour	Portland Bay<br>Portland Harbour<br>Sandsfoot Bay, under Sandsfoot Castle<br>Ledges between Sandsfoot castle and Byng Cliff<br>Castle Cove	
South Devon	Sidmouth	Sidmouth<br>Sidmouth (Ladefoot Rocks)	
South Devon	Teignmouth	Teignmouth<br>Shaldon	
South Devon	Torbay	Torquay<br>Corbyns Head<br>Torquay (Livermead Rocks)<br>Paignton<br>Preston Beach (Paignton)<br>Goodrington (Middlestone to Elberry Cove)<br>Tor Abbey area<br>Tor Abby Rocks<br>Torbay	The large number of historical records from 'Torbay', for which there is no other detail on specific location, have been combined with records from Torquay and other localities south to Elberry Cove. These include a record from Goodrington (1993) from the Devon Biological Records Centre
South Devon	Brixham	Shoalstone Rocks<br>Shoalstone<br>Berry Head (NBN 2015)	Includes record from a cave at Berry Head
South Devon	Plymouth	Plymouth,<br>Wembury (NBN 2015)<br>Firestone Bay (NBN 2015)<br>Adurn Point (NBN 2015)	
Cornwall	Eddystone Lighthouse	Eddystone Lighthouse	Price et al. (1979) were very sceptical about this record due to high wave exposure, however although it was accepted this site has not been re-visited due to remoteness of location.

ESM Table 2. Two-Way ANOVA on frond lengths at Sites Colonels Hard (Sheltered) and Ethel Point (Exposed) at Bembridge, Isle of Wight in 2013 and 2014. Data shows significant differences between main effects and a significant Site:Year interaction. Although there were clear differences in mean frond lengths between years at Ethel Point, differences at Colonels hard were non-significant (>0.05).

	Df	Sum Sq	Mean Sq	F	P
Site	1	23590	23590.2	134.14	<0.001***
Year	1	2399	2399	13.64	<0.001***
Site :Year	1	4307	4307	24.49	<0.001***
Residuals	1702	299319	175.9		

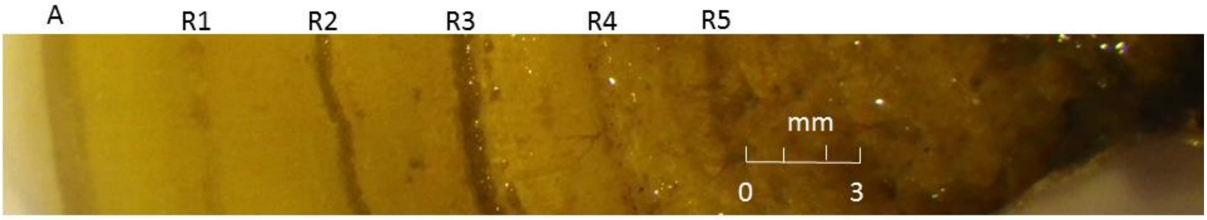

ESM Figure 1. Section through frond of *P.pavonica* collected at Bembridge on 30th August 2014 showing numbering of tetrasporangial rings (R) from the apical region (A) at the inrolled frond margin. Early development is visible in Ring 1. Mature tetraspores are within Ring 3. Spore release has occurred in Rings 4 & 5. (6 -7 rings just visible by eye on this 44mm length frond).
